# Common polymorphisms of calpain-10 and the risk of Type 2 Diabetes in a Tunisian Arab population: a case-control study

**DOI:** 10.1186/1471-2350-11-75

**Published:** 2010-05-15

**Authors:** Intissar Ezzidi, Amira Turki, Safia Messaoudi, Molka Chaieb, Maha Kacem, Ghada M Al-Khateeb, Touhami Mahjoub, Wassim Y Almawi, Nabil Mtiraoui

**Affiliations:** 1 Research Unit of Biology and Genetics of Hematological and Auto-immune diseases, Faculty of Pharmacy of Monastir, University of Monastir, Tunisia; 2 Higher Institute of Biotechnology of Monastir, University of Monastir, Tunisia; 3 Endocrinology and Diabetic Service, CHU Farhat Hached of Sousse, Tunisia; 4 Nephrology and Internal Medicine Service, CHU Fatouma Bourguiba, Monastir, Tunisia; 5 Dept. Medical Biochemistry, Arabian Gulf University, Manama, Bahrain

## Abstract

**Background:**

Genetic variations in the calpain-10 gene (*CAPN10*), in particular the at-risk diplotype (112/121), were previously implicated with increased risk of type 2 diabetes (T2D).

**Methods:**

We examined the association of *CAPN10 *UCSNP-43 (rs3792267), UCSNP-19 (rs3842570), and UCSNP-63 (rs5030952) SNPs with T2D in 917 Tunisian T2D patients and 748 non-diabetic controls. *CAPN10 *genotyping was done by PCR-RFLP.

**Results:**

Enrichment of UCSNP-19 2R (minor) allele and 2R/2R genotype was found in T2D patients; the allele and genotype distribution of UCSNP-43 and UCSNP-63 alleles and genotypes were not significantly different between patient groups and non-diabetic control subjects. Regression analysis demonstrated progressive increases in T2D risk in 3R/2R [OR (95% CI) = 1.35 (1.08 - 1.68)] and 2R/2R [OR (95% CI) = 1.61 (1.20 - 2.18)] genotypes. Of the six haplotypes detected, enrichment of haplotype 111 (UCSNP-43/UCSNP-19/UCSNP-63) was seen in patients (*Pc *= 0.034); the distribution of the other haplotypes was comparable between patients and control subjects; neither haplotype 211 nor haplotype 212 was observed. Furthermore, the frequency of all *CAPN10 *diplotypes identified, including the "high-risk diplotype (112/121) reported for Mexican-Americans and Northern Europeans, were comparable between patients and controls.

**Conclusions:**

*CAPN10 *UCSNP-19 variant, and the 111 haplotype contribute to the risk of T2D in Tunisian subjects; no significant associations between *CAPN10 *diplotypes and T2D were demonstrated for Tunisians.

## Background

Type 2 diabetes (T2D) is a metabolic disorder characterized by impaired insulin-stimulated glucose uptake in muscle and fat, altered glucose-induced insulin secretion, and increased hepatic glucose production. Insofar as T2D is triggered by genetic and environmental risk factors, several genome-wide scan studies on different ethnic groups reported linkage at the same or different chromosomes with T2D [1,2]. Of the diabetes-related genes, fine mapping and positional cloning suggested that the calpain-10 (*CAPN10*) gene might serve as an important T2D susceptibility gene [3,4]. Similar to other calpains, CAPN10 consists of an isoform-specific large subunit and a common small subunit, and was shown to function as intracellular calcium-dependent cysteine proteases in calcium-regulated signaling pathways [5]. CAPN10 is expressed at the mRNA and protein levels by several tissue types, with different mRNA isoforms reported, in particular those involved in the regulation of glucose homeostasis, such as pancreatic β islet cells, liver, skeletal muscle, and adipocytes [6,7].

*CAPN10 *is located on chromosome 2q37.3, and comprises 15 exons spanning 31 kb, and encodes a 672 amino-acid intracellular protease. Several case control and association studies indicated that polymorphisms in *CAPN10 *are associated with the development of T2D and insulin resistance, more so in obese patients with an earlier age of disease onset [6,8-10]. These include the at-risk UCSNP-43 G/A variant located in the third intron, UCSNP-19 2R (two 32-bp repeats)/3R (three 32-bp repeats) present in intron 6, and UCSNP-63 C/T variant within *CAPN10 *intron 13 [7,10]. This contribution of the *CAPN10 *variants to T2D pathogenesis was highlighted by the findings that select UCSNP-43/UCSNP-19/UCSNP -63 haplotypes (112 and 121) were associated with heightened risk of T2D in select ethnic groups, and as at-risk dipolotypes (haplotype combinations) were reported for many populations [10-13]. The reported association of UCSNP-43/UCSNP-19/UCSNP -63 112/121 diplotype with increased T2D risk in Mexicans Americans, German and Finnish populations exemplified this association [10,11].

A number of studies from diverse populations have confirmed the initial findings [6,14], while some did not [15,16]. Others showed a trend towards significance, but were underpowered to detect a meaningful effect [17]. We previously reported on the association of select variants of the recently identified T2DM candidate genes with T2D in Tunisia [18]. The aim of this study was to investigate whether UCSNP-43, UCSNP-19, and UCSNP-63 *CAPN10 *variants, alone or at-risk haplotype and haplotype combination (diplotype] were associated with T2D in a group of Tunisian T2D patients and non-diabetic control subjects.

## Methods

### Study population

A total of 917 consecutive unrelated adult patients with documented medical records of T2D were recruited from outpatient endocrinology clinics in Southern, Central and Northern Tunisia by referral from the treating physician, after obtaining initial verbal consent to participate in the study. In addition, 748 control subjects from the same area as the patients, and comprising blood donors, healthy volunteers, or hospital/university staff members were enrolled in this study. Controls were frequency-matched with patients according to age (*P *= 0.160), ethnic origin (all of Arab descent), and gender (*P *= 0.126), and had to meet the following conditions: normoglycemia (fasting glucose < 5.6 mmol/L) and no personal or first-degree history of diabetes. Diabetes diagnosis was based on clinical features as per World Health Organization criteria. None of the patients had ketoacidosis, and initial T2D treatment comprised diet and/or oral anti-diabetic drugs; patients who required insulin had been treated with oral drugs for at least two years (Table 1). The patients had a mean age of 59.3 years (range 35 - 86 years), BMI of 29.0 kg/m^2 ^(range 18.4 - 39.2 kg/m^2^), and average T2D duration of 12.6 years (range 5 - 36 years), with 330 patients (36.0%) reporting positive family history of diabetes. The control subjects had a mean age of 58.7 years (range 45 - 88 years), and mean BMI of 23.5 kg/m^2 ^(range 18.2-27.0 kg/m^2^). None of the control subjects was taking regular medication, including slimming diet, 6 months prior to inclusion into the study.

**Table 1:** Demographic and Clinical Characteristics of Study Subjects

Characteristic	T2D Patients (917)	Controls (748)	*P*
**Gender (M:F)**	422:495	373:375	0.126
**Mean Age (years)**	59.3 ± 10.9	58.7 ± 8.7	0.160
**Mean B.M.I. (kg/m^2^)**	27.7 ± 4.3	23.5 ± 2.2	< 0.001
**Systolic BP (mmHg)**	140.7 ± 27.0	121.6 ± 14.4	< 0.001
**Diastolic BP (mmHg)**	81.9 ± 12.6	77.9 ± 10.5	< 0.001
**Hypertension**^***2***^	420 (45.8)	86 (18.0)	<0.001
**Fasting glucose (mmol/L)**	12.8 ± 5.3	5.1 ± 0.6	< 0.001
**HbA1c (%)**	9.6 ± 3.9	4.5 ± 1.4	< 0.001
**Age at disease onset (years)**	46.7 ± 10.9	N/A	N/A
**T2D Duration (years)**	12.6 ± 6.3	N/A	N/A
**Total cholesterol (mmol/L)**	5.3 ± 1.4	4.7 ± 1.2	< 0.001
**Triglycerides (mmol/L)**	1.9 ± 1.3	1.2 ± 0.6	< 0.001
**HDL (mmol/L)**	1.1 ± 0.3	1.2 ± 0.4	< 0.001
**LDL (mmol/L)**	3.8 ± 1.4	2.8 ± 1.8	< 0.001
**Urea (mmol/L)**	7.9 ± 4.8	5.6 ± 2.1	< 0.001

1. Student *t-*test for continuous variables, chi square for categorical variables.

2. Defined as BP reading ≥ 145/90 mmHg, and/or use of anti-hypertension medication.

Blood pressure (BP; right arm) was measured twice, using mercury sphygmomanometer with participants in the sitting position following a 5 min rest; the mean of two readings measured 1 minute apart was adopted. Hypertension was determined as BP readings of 145/90 mmHg or higher, and/or use of antihypertensive medications. Demographic details were obtained for all subjects, which included age, gender, BMI, age at onset and duration of diabetes, first-degree family history of diabetes, history of chronic diabetes complications, and treatment of diabetes. The historical information was verified from the clinic records where available. Written informed consent was obtained from all participants, and the study was carried out in accordance with the guidelines of the Helsinki Declaration of 1975, and was approved by the University of Monastir Ethics Committee. Venous blood samples were collected after an overnight fast for measuring plasma glucose, HbA1c, and serum lipids.

### CAPN10 Genotyping

Genomic DNA was isolated from the from the leukocyte-rich buffy coat layer of peripheral venous blood using a QIAamp DNA Blood Mini Kit (Qiagen, Valencia, CA). The study groups were genotyped for the *CAPN10 *UCSNP-43, -19, and -63 polymorphisms as previously described [19,20]. The insertion-deletion UCSNP-19 variant (rs3842570) was genotyped by PCR using the following primers, (forward) 5'-GTT TGG TTC TCT TCA GCG TGG AG-3' and (reverse) 5'-ATG AAC CCT GGC AG G GTC TAA G-3'. PCR fragments were separated on a 4% Nusieve agarose gel; the two 32 bp repeats 2R allele and the three 32-bp repeats 3R allele visualized as155 bp and 187 bp, respectively. UCSNP-43 (rs3792267) was genotyped using the forward primer 5'-CAC GCT TGC TGT GAA GTA ATG C-3', and the reverse primer, 5'-CTC TGA TTC CCA TGG TCT GTA G-3', and subsequent digestion with *Nsi*I (Promega, Madison, WI); the UCSNP-43 alleles seen as 144 bp (G allele) 121 + 23 bp fragments (A allele). UCSNP-63 (rs5030952) was genotyped using the forward primer 5'-AGC ACT CCC AGC TCC TGA TC-3' and the reverse primer 5'-AAG GGG GGC CAG GGC CTG ACG GGG GTG GCG-3', and digestion with *Hha*I (Promega); the UCSNP-63 alleles seen as 162 bp (C allele) and 192 bp fragment (T allele).

All genotyping was carried out in the same laboratory (University of Monastir), and was done in blinded fashion (case/control sample status). Inter-laboratory quality controls comprised independent genotyping of a number of case and control samples by personnel unaware of the phenotype of the samples. Genotyping call rate exceeded 99%, with no significant differences between cases and control samples.

### Statistical Analysis

Statistical analysis was performed on SPSS v.17.0 software (SPSS Inc., Chicago, IL). Data were expressed as mean ± SD for continuous variables that were normally distributed, or as percentages of total for categorical variables. Pearson χ^2 ^test were used to assess inter-group significance, and Student's *t*-test was used to determine differences in means. Allele frequencies were calculated by the gene-counting method, and differences in the allele and genotype frequencies were tested by χ^2 ^test.

Haplotype estimation and determination of diplotype frequencies were done by the expectation maximization (EM) method using HPlus 2.5 software, which is an iterative method that alternates between performing an expectation (E) step (expectation of the log likelihood with respect to the current estimate of the distribution for the latent variables), and a maximization (M) step, which computes the parameters which maximize the expected log likelihood found in the E step. Univariate and multivariate regression analysis were determined using HPlus 2.5 and HAPStat; results being expressed as *p *value, odds ratio (OR) and 95% confidence intervals (CI). Statistical significance was set at *P *< 0.05. The linkage disequilibrium among *CAPN10 *SNPs were calculated by an expectation-maximization algorithm and haplotype frequencies were estimated by LDA v.1.0 software [21].

## Results

### Study Subjects

The demographic and clinical characteristics of T2D patients and controls (Control/Case ratio = 0.82) are shown in Table 1. Patients and control subjects were matched for gender, age, ethnic origin (all Arab subjects), and geographical origin (all originating from Central Tunisia); significant differences were noted between the two groups with regards to BMI (*P *< 0.001), blood pressure (*P *< 0.001), lipid profile (*P *< 0.001), urea (*P *< 0.001), and fasting glucose (*P *< 0.001) and HbA1c levels (*P *< 0.001). T2D duration was 12.6 years (range: 5 - 36 years), with an average age at T2D onset of 46.7 ± 10.9 years.

### CAPN10 Allele and Genotype Frequencies

The genotype frequencies of UCSNP-43 (*P *= 0.291; χ^2 ^= 1.12), UCSNP-19 (*P *= 0.228; χ^2 ^= 1.45), and UCSNP-63 (*P *= 0.506; χ^2 ^= 0.44) were in Hardy-Weinberg Equilibrium. The distribution of UCSNP-43 genotypes (G/G, *P *= 0.255; G/A, *P *= 0.332, and A/A, *P *= 0.518) or minor allele frequency (MAF) (*P *= 0.359), and UCSNP-63 genotypes (C/C, *P *= 0.702; C/T, *P *= 0.895, and T/T, *P *= 0.530) or MAF (*P *= 0.673) was similar between patients and controls (Table 2). The distribution of UCSNP-19 genotypes (3R/3R, *P *= 0.001, 2R/2R, *P *= 0.020), and MAF (*P *= 0.007), were significantly different between patients and controls (Table 2). Homozygosity for the UCSNP-19 2R was associated with increased body weight among patients (OR = 2.17; 95% CI = 1.33 - 3.51) (data not shown). The association of UCSNP-19 genotypes with increased T2D risk was confirmed by regression analysis. Taking homozygous wild-type genotype as reference (OR = 1.00), regression analysis demonstrated progressive increases in T2D risk in 3R/2R [*P *= 0.008; OR (95% CI) = 1.35 (1.08 - 1.68)] and 2R/2R [*P *= 0.002; OR (95% CI) = 1.61 (1.20 - 2.18)] genotypes (Table 3). The association of homozygous UCSNP-19 2R/2R [*P *= 0.044; aOR (95% CI) = 1.40 (1.01 - 1.93)], but not 3R/2R [*P *= 0.923; aOR (95% CI) = 1.02 (0.66 - 1.58)] genotype with changes in BMI remained significant after controlling for age, gender, BMI, hypertension, and lipid profile (Table 3).

**Table 2:** UCSNP-43, UCSNP-19 and UCSNP-63 Allele and Genotype Distribution

SNP	Allele/Genotype	Patients (917)	Controls (748)	***P ***^***1***^
***UCSNP-43***	MAF^*2 *^(**A**)	0.088	0.101	0.359
	G/G	759 (0.828)	602 (0.805)	
	G/A	155 (0.178)	141 (0.189)	0.343
	A/A	3 (0.003)	5 (0.007)	
***UCSNP-19***	MAF (**2R**)	0.464	0.404	**0.007**
	3R/3R	247 (0.269)	258 (0.345)	
	3R/2R	489 (0.533)	376 (0.503)	**0.001**
	2R/2R	181 (0.197)	114 (0.152)	
***UCSNP-63***	MAF (**T**)	0.162	0.170	0.673
	C/C	638 (0.696)	513 (0.686)	
	C/T	261 (0.285)	216 (0.289)	0.704
	T/T	18 (0.020)	19 (0.025)	

1. Pearson chi square test

2. MAF = minor allele frequency.

**Table 3:** Regression Analysis on T2D Risk Associated with CAPN10 Polymorphisms ^*1*^

		Univariate		Multivariate	
*SNP*	Genotype	*P*	OR (95% CI)	*P*	aOR ^*2 *^(95% CI)
***UCSNP-43***	G/G	0.647	1.00 (Reference) ^*3*^	0.809	1.00 (Reference) ^*3*^
	G/A	0.573	1.52 (0.35 - 6.53)	0.827	1.20 (0.24 - 6.12)
	A/A	0.492	1.66 (0.39 - 7.03)	0.708	1.36 (0.27 - 6.85)
***UCSNP-19***	3R/3R	**0.003**	1.00 (Reference) ^*3*^	0.084	1.00 (Reference) ^*3*^
	3R/2R	**0.008**	**1.35 (1.08 - 1.68)**	0.923	1.02 (0.66 - 1.58)
	2R/2R	**0.002**	**1.61 (1.20 - 2.18)**	**0.044**	**1.40 (1.01 - 1.93)**
***UCSNP-63***	C/C	0.714	1.00 (Reference) ^*3*^	0.704	1.00 (Reference) ^*3*^
	C/T	0.463	1.29 (0.66 - 2.52)	0.722	1.19 (0.45 - 3.17)
	T/T	0.417	1.31 (0.68 - 2.54)	0.920	1.05 (0.39 - 2.85)

1. Study subjects comprised 917 cases and 748 age-, gender-, and origin-matched controls subjects.

2. aOR = adjusted OR; covariates included age, gender, BMI, hypertension, and lipid profile.

3. Reference group being minor allele non-carriers.

### Haplotype and Diplotype Distribution

Linkage disequilibrium (LD) analysis for the *CAPN10 *variants, defined by the delta coefficient, showed a weak negative LD between these SNPs. LD among the three markers (*r*^2^) ranged from 0.001 to 0.083 in T2D subjects and from 0.002 to 0.076 in non-diabetic controls (Table 4). Six possible *CAPN10 *haplotypes were inferred, and neither haplotype 211 nor haplotype 212 was observed. Enrichment of only haplotype 111 was seen in patient [*Pc *= 0.034; OR (95% CI) = 1.22 (1.06 - 1.41)]; the distribution of the other haplotypes was comparable between unselected patients and control subjects (Table 5). Data from Table 6 showed that the frequency of all *CAPN10 *diplotypes identified, including the "high-risk haplotype (112/121) reported for Mexican-Americans and Northern Europeans, were comparable between patients and controls, even before applying the Bonferroni correction.

**Table 4:** Linkage Disequilibrium Analysis Between *CAPN10 *SNPs ^*1*^

		T2D Patients (n = 917)			Controls (n = 748)		
***Locus 1***	**Locus 2**	**D'**	**r**^**2**^	***P***	**D'**	**r**^**2**^	***P***
							
***UCSNP-43***	**UCSNP-19**	1.000	0.083	<0.001	1.000	0.076	<0.001
***UCSNP-43***	**UCSNP-63**	0.360	0.002	0.190	0.284	0.002	0.314
***UCSNP-19***	**UCSNP-63**	0.057	0.001	0.516	0.170	0.004	0.118

1. Analyzed by LDA v.1.0 software.

**Table 5:** *CAPN10 *Haplotype Analysis

**Haplotype**^***1***^	Patients	Controls	*P*	*Pc*^*3*^	OR (95% CI)
**111**^*4*^	0.392 (719) ^***2***^	0.345 (516) ^***2***^	**0.006**	**0.034**	**1.22 (1.06 - 1.41)**
112	0.072 (132)	0.059 (88)	0.147	0.615	1.24 (0.94 - 1.64)
121	0.368 (675)	0.399 (597)	0.072	0.361	0.88 (0.76 - 1.01)
122	0.080 (147)	0.096 (144)	0.115	0.520	0.82 (0.64 - 1.04)
221	0.078 (143)	0.086 (129)	0.422	0.963	0.90 (0.70 - 1.15)
222	0.010 (18)	0.015 (22)	0.259	0.834	0.66 (0.36 - 1.24)

1. Haplotype frequency determined by the maximum likelihood method.

2. Haplotype frequency (number of haplotypes).

3. *Pc *= corrected *P*, calculated as per the Bonferroni method [*Pc *= 1 - (1 - *P*)^n^)], where n = number of comparisons.

4. *CAPN10 *haplotypes were coded as per the allele at each locus (wild-type = 1, mutant = 2); the first refers to UCSNP-43, the second to UCSNP-19, and the third to UCSNP-63.

**Table 6:** *CAPN10 *Haplotype Combinations

**Diplotype**^***1***^	Patients	Controls	*P*	***Pc ***^***2***^	**OR**^***3***^**(95% CI)**
111/111	132 (0.144) ^***4***^	88 (0.118)	0.133	0.864	1.26 (0.94 - 1.70)
111/112	45 (0.049)	23 (0.031)	0.079	0.684	1.63 (0.97 - 2.67)
111/121	260 (0.284)	193 (0.258)	0.268	0.987	1.14 (0.91 - 1.41)
111/221	76 (0.083)	57 (0.076)	0.683	1.000	1.10 (0.76 - 1.56)
112/112	4 (0.004)	3 (0.004)	0.947	1.000	0.81 (0.22 - 3.02)
112/121	115 (0.125)	87 (0.116)	0.624	1.000	1.09 (0.81 - 1.46)
112/221	28 (0.031)	32 (0.043)	0.230	0.974	0.70 (0.42 - 1.18)
121/121	132 (0.144)	131 (0.175)	0.095	0.753	0.79 (0.61 - 1.03)
121/221	35 (0.038)	39 (0.052)	0.194	0.951	0.72 (0.45 - 1.15)
221/221	3 (0.003)	5 (0.007)	0.519	1.000	0.49 (0.13 - 1.98)
112/122	10 (0.011)	7 (0.009)	0.946	1.000	1.17 (0.45 - 2.93)
121/122	58 (0.063)	62 (0.083)	0.148	0.894	0.75 (0.52 - 1.08)
122/122	3 (0.003)	8 (0.011)	0.120	0.833	0.30 (0.10 - 1.16)
122/221	16 (0.017)	13 (0.017)	0.859	1.000	1.00 (0.48 - 2.06)

1. *CAPN10 *haplotypes were coded as per the allele (wild-type = 1, mutant = 2) at each locus; the first refers to UCSNP-43, the second to UCSNP-19, and the third to UCSNP-63.

2. *Pc *= corrected *P*, calculated as per the Bonferroni method [*Pc *= 1 - (1 - *P*)^n^)], where n = number of comparisons.

3. Calculated according to Woolf's method, for specific patient *vs*. control diplotype carriers.

4. Number (frequency).

## Discussion

*CAPN10 *was the first T2D candidate gene identified through genome-wide screening and positional cloning [3,8], and increasing evidence has implicated contribution of *CAPN10 *gene variants in the risk of T2D [9,10,13]. Specific *CAPN10 *variant (UCSNP-43) and at-risk haplotype combination (112/121) defined by UCSNP-43, -19, and -63 polymorphisms, reportedly confers increased risk of T2D in some but not all populations [4,9,11,13,15]. Insofar as the Tunisian population (est. 9.6 million) is characterized by a high ethnic diversity, brought about by the admixture of the native Berbers (descendants of the Mesolithic Capsian populations) with principally the invading Arabs in the 7^th^-8^th ^Century A.D. [22], we limited our study group to only Tunisians of Arab descent, so as to minimize the inherent problems associated with multi-ethnic genetic studies.

We previously investigated the contribution of some candidate gene variants identified through GWAS *(TCF7L2, HHEX, GCK, ENPP1 and KCNJ11) *to T2D in Tunisian; only *TCF7L2 *(rs7903146) was significantly associated with T2D in Tunisians [18], thereby demonstrating ethnic contribution of the association of specific gene variants to T2D pathogenesis. In this case-control study, we examined the contribution of UCSNP-43, -19, and -63 *CAPN10 *variants on T2D risk in Tunisians of Arab origin, which were selected in view of previous reports linking them with T2D [4,7,14], insulin resistance [15], obesity [7,9,15], or with altered regulation of *CAPN10 *gene expression in Tunisian and other populations. In the population studied, we could not confirm the previously described haplotype associated with T2D (112/121) in Mexican-Americans and in northern-European populations [4,9-11]. UCSNP-19 was shown to affect the susceptibility to T2D, evidenced by enrichment of the 2R allele (*P *= 0.007; OR = 1.15) and homozygous 2R/2R genotype (*P *= 0.002; OR = 1.61) in T2D patients.

In agreement with previous reports, UCSNP-19, but UCSNP-43 and UCSNP-63 was associated with heightened T2DM risk and increased BMI [6], without ruling out a role for UCSNP-43 and UCSNP-63 in the development of T2DM-related traits (insulin resistance, altered lipid metabolism), as was suggested [7,15]. Previous studies implicated UCSNP-19 with altered insulin sensitivity (HOMA-IR index) in Northern European [17] and Spanish [23], but not Scandinavian [15] or Finnish [7] subjects. Our findings were in agreement with a recent small study in Southern Tunisia (Djerba Island) involving 162 T2D patients and 110 control subjects of mixed Arab and non-Arab (Berber) ancestry, in which UCSNP-19 was associated with T2D only in the Arab sub-group [24], and in apparent disagreement with another study in Central Tunisia (Sfax), in which UCSNP-43, but not UCSNP-19, was over-represented in T2D patients [25]. While explanations for the discrepancies remain to be seen, they most likely reside in inadequate statistical power in the study of Kifagi (226 patients and 206 controls), and in differences in subjects' selection (gender distribution, duration of diabetes, BMI status), which in turn may have overestimated the association of UCSNP-43 with T2D.

In the Tunisian population studied herein, the most frequent haplotypes were 111 in patients (OR = 1.22) and 121 among controls (OR = 0.88), while haplotype 112 was detected at low frequencies among controls (0.059) and patients (0.072). Haplotype 112 is common among Africans and Asians, but is infrequent in Caucasians, while haplotype 121 is infrequent in Africans, but common among Europeans [8,26]. This relatedness of North African Tunisians to Europeans in the distribution of the *CAPN10 *haplotypes, more so than Africans, may be explained by the admixture of indigenous African inhabitants (Berbers) with Phoenicians (ancestors of present-day Lebanese), followed by successive migration of Muslims from Arabian Peninsula, Turks (Ottoman rule), and recently Europeans. The positive association of haplotype 111 with T2D among Tunisians was reminiscent of previous results linking haplotype 111 with increased T2D risk in Koreans [27], and with altered insulin sensitivity in Spanish subjects [23]. The reason for discrepancies in the association of specific *CAPN10 *haplotypes, especially 111 haplotype rather than the anticipated 121 haplotype with T2D remains speculative at this stage. The heterogeneity of *CAPN10 *in the magnitude of LD between *CAPN10 *variants is likely attributed to ethnic differences, coupled with sample size differences, and to the failure to control for possible confounding variables by some of the studies. Collectively, this may have modulated potential effects of *CAPN10 *gene variants on T2D.

Since the first report of Horikawa linking specific *CAPN10 *at-risk haplotype combination (112/121) (defined by UCSNP-43, -19 and -63), with higher risk of T2D in Mexican-Americans and Europeans [10], a number of studies performed on diverse populations yielded often inconsistent association of *CAPN10 *diplotypes with T2D risk [9,10]. These haplotype combinations included 111/121 in Koreans [27], 111/221 in Northern Europeans [17], 112/221 in Chinese [12], and 121/121 in pan-Eurpoean populations [9]. We did not identify specific T2D at-risk CAPN10 haplotype combination, including the 112/121 diplotype, which was present at comparable frequencies among healthy controls and T2D patients.

Our findings were in agreement with those reported for the Scandinavians [15], Koreans [27], and Mexicans [11] and in meta-analysis conducted in both population-based and family-based association studies [26] and in prospective cohort study of multi-ethnic American postmenopausal women [28], in which 112/121 diplotype did not influence the risk of T2D. It was of interest to note the association of 121/221 diplotype with increased risk of T2D among Tunisians (South East Tunisia) [25]. While the frequency of this diplotype was comparable between our healthy control group (0.052) and their control cohort (0.05), marked difference its distribution among T2D patients was seen between our (0.038) and their (0.108) T2D patient groups. These discrepancies may be explained by differences in sample size (226 patients and 206 controls in Kifagi study vs. 917 patients and 748 controls in our study), and thus statistical power, differences in patient characteristics (BMI, hypertension status, and T2D duration), and data presentation. Future large-scale association and functional studies are needed to confirm or argue against the association of a particular *CAPN10 *haplotype combination with T2D risk, after controlling for the ethnic/geographical factor.

## Conclusion

In conclusion, we were not able to replicate the association between specific *CAPN10 *alleles identified in earlier studies and T2D in Tunisians of Arab ancestry. The difference in the association between *CAPN10 *variants with increased risk of T2D between populations (Mexican Americans, northern-Europeans, Pima Indians) may be attributed to the presence of multiple susceptibility alleles at *CAPN10 *locus, to different linkage disequilibrium patterns of between these variants (and hence haplotypes and haplotype combinations), racial/ethnic differences in the distribution of *CAPN10 *variants, multiple hypothesis testing, and to inadequate statistical power in a number of these studies, which has likely overestimated this genetic association. However, our study has some limitations. It was limited to three SNPs which were selected based on previous reports, leaving the possibility of the contribution of other *CAPN10 *SNPs to T2DM pathogenesis among Tunisians to be addressed, together with the speculation as to if the three SNPs analyzed were sufficient to capture the genetic variability in Tunisian Arabs. In addition, we did not assign a functional aspect to the *CAPN10 *variants and haplotypes included, and it was limited to a specific ethnic group (North African Tunisian Arabs). These, coupled with the potential linkage of *CAPN10 *polymorphisms investigated with other *CAPN10 *or nearby gene polymorphisms, necessitates large population-based follow-up studies for better understanding of the contribution that *CAPN10 *to T2D risk.

## Abbreviations

CAPN10: Calpain 10; BMI: Body Mass Index; CI: Confidence Interval; MAF: Minor Allele Frequency; OR: Odds Ratio; PCR: Polymerase Chain Reaction; SNP: Single Nucleotide Polymorphism; T2D: Type 2 Diabetes.

## Competing interests

The authors declare that they have no competing interests.

## Authors' contributions

IE participated in the design of the study, carried out the SNP genotyping and the analyses of the genotype data, and contributed to the statistical analyses and the drafting of the manuscript. AT and SM participated in the SNP genotyping and some of the genetic analyses. MC and MK coordinated the patients' recruitment. GMAK and TM contributed to the manuscript editing. WYA and NM contributed to the design and coordination of the study, to the statistical analyses and drafted the manuscript. All authors read and approved the final manuscript.

## Pre-publication history

The pre-publication history for this paper can be accessed here:

http://www.biomedcentral.com/1471-2350/11/75/prepub
